# Choosing wisely: practical considerations on treatment efficacy and safety of asthma in the elderly

**DOI:** 10.1186/s12948-015-0016-x

**Published:** 2015-06-22

**Authors:** Nicola Scichilone, Maria T Ventura, Matteo Bonini, Fulvio Braido, Caterina Bucca, Marco Caminati, Stefano Del Giacco, Enrico Heffler, Carlo Lombardi, Andrea Matucci, Manlio Milanese, Roberto Paganelli, Giovanni Passalacqua, Vincenzo Patella, Erminia Ridolo, Giovanni Rolla, Oliviero Rossi, Domenico Schiavino, Gianenrico Senna, Gundi Steinhilber, Alessandra Vultaggio, Giorgio Canonica

**Affiliations:** Department of Medicine, University of Palermo, via Trabucco 180, 90146 Palermo, Italy; Interdisciplinary Department of Medicine, Unit of Geriatric Immunoallergology, University of Bari, Bari, Italy; Lung Function Unit, Department of Public Health and Infectious Diseases “Sapienza”, University of Rome, Rome, Italy; Respiratory Diseases & Allergy Clinic, University of Genoa, IRCCS AOU San Martino-IST, Genoa, Italy; Pneumology Unit, Department of Medical Sciences, University of Turin, AOU San Giovanni Battista, Torino, Italy; Allergy Unit, Verona University and General Hospital, Verona, Italy; Department of Medical Sciences “M. Aresu”, University of Cagliari, Cagliari, Italy; Department of Clinical and Experimental Medicine - Respiratory Medicine & Allergy, University of Catania, Catania, Italy; Departmental Unit of Allergology-Clinical Immunology & Pneumology, Fondazione Poliambulanza, Brescia, Italy; Centre of Excellence DENOTHE, Dept. of Experimental and Clinical Medicine, Units of Immunoallergology Azienda Ospedaliero-Universitaria Careggi, Florence, Italy; Struttura Complessa di Pneumologia, ASL2 Savonese, Savona, Italy; Laboratory of Immunology and Allergy, Department of Medicine and Sciences of Aging, University of G. d’Annunzio, Chieti Scalo, Italy; Division of Allergy and Clinical Immunology, ASL SALERNO, Hospital of Battipaglia, 84100 Salerno, Italy; Department of Clinical and Experimental Medicine, University of Parma, Parma, Italy; Allergologia e Immunologia Clinica, AO Ordine Mauriziano & University of Torino, Torino, Italy; Units of Immunoallergology Azienda Ospedaliero-Universitaria Careggi, Florence, Italy; Università Cattolica del Sacro Cuore, Policlinico A.Gemelli, Rome, Italy; Division of Pneumology, Spedali Civili di Brescia, Brescia, Italy

**Keywords:** Aging, Asthma, Allergy, Therapy

## Abstract

The prevalence of asthma in the most advanced ages is similar to that of younger ages. However, the concept that older individuals may suffer from allergic asthma has been largely denied in the past, and a common belief attributes to asthma the definition of “rare” disease. Indeed, asthma in the elderly is often underdiagnosed or diagnosed as COPD, thus leading to undertreatment of improper treatment. This is also due to the heterogeneity of clinical and functional presentations of geriatric asthma, including the partial loss of reversibility and the lower occurrence of the allergic component in this age range. The older asthmatic patients are also characterized the coexistence of comorbid conditions that, in conjunction with age-associated structural and functional changes of the lung, may contribute to complicate the management of asthma. The current review addresses the main issues related to the management of allergic asthma in the geriatric age. In particular, the paper aims at revising current pharmacological and non pharmacological treatments for allergic asthmatics of advanced ages, primarily focusing on their safety and efficacy, although most behaviors are an arbitrary extrapolation of what has been tested in young ages. In fact, age has always represented an exclusion criterion for eligibility to clinical trials. Experimental studies and real life observations specifically testing the efficacy and safety of therapeutic approaches in allergic asthma in the elderly are urgently needed.

## Review

The world population is ageing, and life expectancy (meaning the number of years an individual can expect to live) is steadily increasing. Because of increased longevity, the proportion of individuals aged 65 years and older (referred to as the elderly) is growing worldwide. Given these demographic changes, the impact of asthma is expected to rise in the next years. The management of asthma in the elderly follows international guidelines that apply to all ages, although most recommendations are an arbitrary extrapolation of what has been tested in younger subjects. In fact, age has always represented an exclusion criterion for eligibility to clinical trials. Many specific factors affect treatment of the elderly. They often undertake a number of medications as they frequently suffer from different diseases and comorbidities. Polypharmacy increases the risk of low adherence and of interactions between different drugs. Furthermore elderly patients may present cognitive dysfunction that results in a decreased memory, which in turn affects the compliance to the treatments. The changes in body composition and metabolism that characterize aging process should also be carefully taken into account. The pharmacological effect of systemic drugs may be affected by the reduced activity of kidney and liver and by the decrease in muscle mass, fat and body water. In elderly individuals with respiratory allergies, the ‘course’ of the disease is considered too advanced, and therefore the therapeutic value of specific immunotherapy is considered very limited. Physicians often face the requests of patients who ask for complementary/alternative medicines (CAMs). Thus, it is urgent to become aware of the efficacy and contraindication of CAMs in order to provide patients with scientifically-supported information, especially in older populations. Strategies to increase physical and sports activity participation among older people should include the awareness of the benefits and minimize the perceived risks of physical activity. Despite advances in the management of asthma independently of age, there is an urgent need for targeted, disease-modifying asthma treatments. It is necessary to clearly identify clinical phenotypes to achieve optimal treatment of elderly patients with asthma. It is clear that the delivery of biologics or advanced immunotherapy requires a special attention in the elderly, where comorbidities are often present.

The current article undergoes the main aspects of pharmacological and non pharmacological approaches to asthmatic subjects of advanced ages, with special focus on safety issues, and highlights the practical considerations to be taken into account when managing elderly asthmatics in clinical settings.

### Inhaled treatment

#### Corticosteroids

Inhaled corticosteroids (ICS) are widely used in the treatment of patients with asthma, in that, they represent the cornerstone of the pharmacological management of the disease. However, high-dose regimens and long-term use of ICS may be associated with increased risk of side-effects, which are mostly important in the elderly populations. The GINA guidelines [1] clearly state that asthma treatment in the elderly is complicated by several factors, such as the reduced coordination between activation of the device and inhalation of the drug, which can affect the lung deposition on one hand (thus reducing the efficacy), and can increase the oral deposition on the other hand (thus causing local and systemic adverse events). In addition, the increased number of comorbidities and their associated symptoms and treatment may interact to various extent with the pharmacological treatment for asthma, potentially leading to serious side effects.

The pharmacokinetic and pharmacodynamics features of the ICSs are influenced by several factors, such as the particle size and the formulation of the aerosol. These factors can affect the systemic bioavalaibility, which is responsible for the potential suppression of the hypothalamic-pituitary-adrenal axis. The bioavailability is also influenced by the protein binding and the process of bioactivation by first pass metabolism of the liver. All these passages can be altered in the most advanced ages, and should be taken into account, by using the minimum efficacy dose of ICS.

With no doubt, the most serious potential side effect is the increased incidence of pneumonia, which has been observed in patients with COPD both in controlled clinical trials and case–control analyses [2]. There is evidence that the occurrence of pneumonia is associated with the use of ICS also in asthma [3]. Because of the long-term use of ICSs, safety concerns have been raised with regard to osteoporosis and risk of fractures [4], although the occurrence is much lower in comparison with the use of systemic corticosteroids. The clinical implications for elderly asthmatics may not be trivial, since these subjects will likely continue to be exposed to high doses of ICS over many years.

Patients and physicians are often concerned about the use of ICSs and the occurrence, or worsening, of diabetes. Suissa and collaborators [5] found that ICS use was associated with a 34% increase in the risk of incident diabetes, defined as initiation of anti-diabetic medications, in a dose–response fashion. Moreover, in patients already treated for diabetes with oral hypoglycemic agents, the risk of progression to insulin also increased significantly with the use of ICS. With regard to the occurrence of glaucoma and cataract, the available literature has not confirmed the link with the chronic use of ICS [6,7], although a careful check for ocular abnormalities and the monitoring of ocular pressure is always recommended. Local side effects, such as oral, pharyngeal and even esophageal candidiasis are common adverse effects of ICS. However, little is known about the prevalence in the elderly. It is logical to assume that factors such as the patient’s inhalation technique, patterns of inhalation and peak inspiratory flow, all of which are variably impaired in elderly patients, can increase the occurrence of these side effects in the most advanced ages [8,9].

#### Beta-2 adrenergic agonists

Beta-2 agonists, both short- and long-acting (SABA and LABA), are widely used in elderly asthmatic patients and their efficacy is well established [8,10]. Aside the valid and prolonged bronchodilator effect, long-acting molecules may also exert a beneficial steroid sparing action if added to moderate dose inhaled corticosteroids instead of increasing the use of the latter. The chronic use of beta-2 agonists can however lead to the onset of tolerance. This has been mainly reported in specific phenotypes of asthma like exercise-induce bronchoconstriction (which shares a prevalent neutrophilic inflammatory pattern with elderly asthma) and in peculiar subpopulations of subjects, despite data in aged patients are not available in literature. Furthermore, responsiveness to this class of drugs may decline with age, due to a beta-adrenergic dysfunction [10]. In addition, clinicians should limit the prescription of SABA to that for rescue therapy, advising any patient using them more than twice weekly to return for reassessment of asthma control.

Besides these general indications, there are certain therapeutic concerns related to the beta-2 adrenergic administration, unique to older patients, which deserve to be addressed. Among these, the most important seems to be the greater probability of adverse effects, in the setting of multiple comorbidities [11]. Hypokalemia, QT prolongation, tachycardia and tremor are the side effects more commonly reported in association with these agents. They are mediated by the systemic drug absorption and are dose dependant. In particular, the incidence of dysrhythmias after the administration of nebulized beta-2-agonists is well recognized and it has been reported to be as high as 65% [12]. However, clinical trials have not specifically addressed the use of SABA and LABA in elderly asthmatic patients. This is unfortunate, since the incidence of ischemic heart disease and the coexistence of other cardiovascular disorders increase with age, and many patients with chronic lung disease are smokers. Furthermore, beta-2 agonists cause a net influx of intravascular potassium into cells with subsequent hypokalemia. Older patients taking diuretics or insulin, as well as those with poor nutritional intake have a greater incidence of hypokalemia and are thus at greater risk of developing this common electrolyte disturbance.

#### Anticholinergics

The use of anticholinergic drugs in elderly should take into consideration the detrimental effect of aging on the parasympathetic activity and the possible occurrence of adverse events. Ipratropium bromide is a short-acting anticholinergic bronchodilator that is routinely used for COPD, and is less commonly used as first-line therapy to treat asthma. However, ipratropium is often prescribed in combination with albuterol for the treatment of acute exacerbations of asthma in emergency rooms. Results of previous research studies showed the benefit of using the combination therapy in adults with acute asthma (mean age 34.3 ± 10.5 years), leading to a decreased rate of hospital admission compared with albuterol alone [13]. Tiotropium has been considered as add-on therapy to ICS and LABA, and the results of randomized controlled trials suggest a significant effect on lung function. However, all trials enrolled adults with an overall mean age of 49 ± 11 yrs, preventing, at present, definitive conclusion about the efficacy of tiotropium in elderly asthmatics [14-16].

Long acting anticholinergic are well tolerated in the elderly. Dry mouth, and unpleasant taste can occur and these adverse events can contribute, in older people, to reduced ability to speak, mucosal damage, denture misfit, poor appetite, malnutrition risk and respiratory infection due to the reduction of antimicrobial activity of saliva. In males, urinary outflow can be observed and a reduction of gastrointestinal motility has been documented in adults. Moreover, anticholinergics increase intraocular pressure and can cause dilatation of pupil and blurred vision. Cardiovascular effects have been deeply explored in COPD patients, and the available results regarding their safety may be considered encouraging. Due to the reduced metabolism and drug elimination in older patients, anticholinergic drugs may induce, in continuous users, mild cognitive impairment. Finally, in 3 patients out 1000 a paradoxical bronchocostriction may occur [17]. On the basis, the use of long acting anticholinergic drugs should be limited to elderly people that remain uncontrolled despite ICS and LABA use, LABA intolerance and ineffectiveness of other therapeutic approaches.

### Systemic treatment

#### Systemic glucocorticoids

Systemic glycocorticoids (mainly administered by oral route) are listed as second line option in GINA guidelines step 5 and at low dose (i.e. < 7.5 mg/die equivalent of prednisone). Their use is limited to adults with poor control and reserved to elderly patients who may have more benefit than side effects, which increase with the dose administered. They are to be administered when flares-up of symptoms develop in the course of well-controlled treatment and when acute emergency occurs, including hospital care. Use of oral steroids in asthma was more liberally prescribed in the past, when the concepts of steroid-resistance and dependence were developed. Nowadays, systemic steroids are the final approch when all other strategies have failed or are not applicable. About 40% of asthmatic patients above 75 yrs of age do not control their asthma, and this is only partly related to cortisonophobia of both patients and doctors. Obesity is another factor predisposing to diminished steroid response, even after adjusting for body weight. When serious life threatening acute episodes occur, or on emergency hospital admission, prednisolone at 1/mg/kg (with maximum of 50 mg) is recommended [18]. From a clinical perspective, it is interesting to point out that oral route (when not contraindicated) is as efficacious as the intramuscular or intravenous routes. Therapy should not exceed seven consecutive days, but no problems arise from abrupt discontinuation for treatments below 14 days. Beyond this time frame, a gradual titration of oral steroids is advisable.

Side effects due to systemic glycocorticoid treatment include glucose intolerance (usually reversible or controlled by treatment), gastrointestinal bleeding (in patients with known disease or gastrectomized for previous ulcers) and blood pressure control (not a main problem since step-up antihypertensive therapy controls the increase). Depression and changes of mood are frequent, especially in the elderly populations, and other side effects such as catharact, glaucoma, osteoporosis and adrenal insufficiency may occur in the long-term treatment. The danger of immunosuppression is not common and it is even less frequent than with inhaled corticosteroids (i.e. candidiasis, tuberculosis). When elderly patients are discharged from hospital, adherence to treatment abruptly ceases and a flare-up of symptoms after discontinuation of systemic steroids is considered a bad prognostic sign and indication of greater severity of asthma [19].

#### Leukotrienes antagonists

There are two different licensed leukotriene (LT) Antagonists (LTRA) for asthma treatment: a) a 5- lipoxygenase enzyme inhibitor named zileuton, and three LT-1 antagonists named montelukast, pranlukast, zafirlukast. In the COMPACT [20] study montelukast showed its ability to spare on ICS dosage doubling ICS to obtain asthma control; while in the IMPACT [21] study montelukast showed its ability to be an alternative to salmeterol in patients treated with fluticasone propionate. When considering this option in the elderly, a recent observational study on asthmatics ≥65 yrs reported that about in a quarter of them montelukast was used as add-on therapy to a LABA/ICS combination [22]. Theoretically, montelukast could be of interest in the treatment of asthma in the elderly, as it could contribute to obtain symptom control by enhancing patients’adherence, frequently reduced in the elderly [23], increasing the efficiency of asthma therapy, by counterbalancing well-known errors in managing inhaler devices, also frequent in the elderly [24], and avoiding ICS and LABA side effects, reported with higher frequency in the elderly [25], and considered a component of the future risk of asthma. It is of interest the ability of LTRA to be an alternative to β2 agonists when hypertension and/or hearth failure and/or chronic ischemic heart disease are present, becoming the first-line drugs for elderly asthmatics with a history of cardiovascular events. Moreover, avoiding a low dose ICS or sparing on its doubling dosage may be useful when comorbidities are present (osteoporosis, diabetes, glaucoma, cataract). In one double blind, randomized, placebo-controlled study on subjects aged >60 yrs with severe asthma, control was improved by adding montelukast to LABA/ICS combination. Subjects experienced reduced exacerbations, asthma symptoms and salbutamol use [26]. The authors hypothesized that these positive effects were due to higher adherence to the once daily oral treatment with added montelukast. However, a retrospective analysys on five clinical trials [27] comparing zariflukast and fluticasone propionate did not show any significant benefit on a population aged >50 yrs. A recent revision of Scichilone et al. [28] on the safety and efficacy of montelukast in the elderly population is reassuring about safety, but is unable to conclude for a superior efficacy of LTRAs in this setting. However, the authors concluded that in the elderly population LTRAs, and particularly montelukast, may represent a more effective strategy in improving asthma given unintentional nonadehrence with inhalation therapy.

#### Theophyllines

Theophylline, a methylxanthine, has been used as a bronchodilator in the treatment of asthma for more than 80 years, and remains a widely prescribed drug, because it is cheap and readily available [29]. Unfortunately, relatively high doses of theophylline are required to obtain a bronchodilator effect (10–20 mcg/ml), and this is associated with high incidence of side effects, mostly due to adenosine antagonism [30]. Theophylline treatment is also limited by its narrow therapeutic range, variable inter-patient pharmacokinetics, and multiple drug interactions. However, in recent years, the interest for theophylline is reborn through the demonstration of its anti-inflammatory activity [29,31]. Actually, even at low therapeutic concentrations (5 mcg/ml) it is able to activate the histone deacetylases, especially when their activity is reduced by oxidative stress, as in smokers [32].

In general, the use of theophylline is limited by its adverse effects, which range from commonly occurring gastrointestinal symptoms to palpitations, arrhythmias, rarely myocardial infarction and seizures. Theophylline is metabolized primarily by the liver, and commonly interacts with other medications. The available data indicate that the clearance of theophylline is reduced by 22-35% in elderly people, and that it is furtherly decreased by concomitant diseases, particularly liver (50% decrease) and heart (50% decrease) disease. Theophylline clearance is also influenced by diet: low carbohydrate/high protein diets, parenteral nutrition, and daily consumption of charcoal-broiled beef increase the clearance and decrease half-life. Curiously, theophylline clearance is increased by 80% in elderly tobacco smokers. However, appropriate studies have not demonstrated geriatric-specific problems that would limit the usefulness of theophylline in the elderly. In conclusion, the main roles of theophylline in asthma of the elderly are in severe disease as an adjunct to ICS and LABA, particularly in conditions of corticosteroids resistance and in smokers [33]. Caution and close monitoring of plasma theophylline concentration are required in the elderly.

The phosphodiesterase (PDE) 4 inhibitor roflumilast is available for COPD treatment, and its use in asthma can be an interesting add-on therapeutic option in severe asthma with frequent exacerbation and neutrophilic inflammation. Its therapeutic use of is limited by side-effects, which are dose-dependent and the range of efficacy/tolerability is narrow. No difference in safety or efficacy was found between older and young patients and no cardiovascular risks emerged in studies [34]. The main limitations are associated with class-specific side effects such as emesis, reported as a dose-limiting side effect, nausea and diarrhea. Weight loss is a major concern, reported in daily clinical settings.

#### Monoclonal antibodies

To date, omalizumab, a monoclonal anti-IgE humanized antibody, is the only specific target therapy available, and recognised as an add-on therapy in severe persistent asthmatics with inadequately controlled symptoms, regardless of age. Despite the high safety profile of omalizumab, an association between the use of omalizumab and the occurrence of hyperglycemia has been recently documented, and related to the sucrose contained in the vials [35]. Taking into account the incidence of diabetes in elderly patients, clinicians must control the blood levels of glucose in asthmatic patients during omalizumab treatment.

Infliximab, a chimeric anti-TNF-α monoclonal antibody (mAb) and etanercept, a soluble TNF-α receptor linked to human Fc of IgG1, showed significant improvements in lung function and in the exacerbation rate, particularly in patients with severe steroid-resistant asthma. However, conflicting efficacy results obtained with other TNF-α blockers have cooled the use of these biologicals in asthmatic patients [36]. The crucial role of IL-5 in eosinophil activation, maturation and survival makes it an interesting drug target. In fact, the inhibition of eosinophil accumulation in the airway wall of asthmatic patients by using mepolizumab and reslizumab, two humanized anti-IL-5 mAbs, and benralizumab, an anti-IL-5 receptor represent novel therapeutic strategies. IL-4 is an established clinical target and a key factor in airway inflammation and IgE synthesis by B cells; however, despite initially promising findings with biologics able to block its functions (pitrakinra, pascolizumab, dupilumab), subsequent trials are needed before its clinical application [37]. In conclusion, other than the anti-IgE mAb omalizumab, novel therapies are currently being explored to overcome the difficulties of severe asthma, even though no specific treatment are dedicated to older asthmatic patients.

### Specific immunotherapy

#### Subcutaneous immunotherapy

Subcutaneous immunotherapy (SCIT) is the historical route of administration and consists of allergen extract injections which can only be performed with a medical observation. The guidelines on the treatment of allergic diseases rarely focus on the elderly population and often ignore this population completely. Several placebo-controlled studies that demonstrated efficacy have included subjects up to 60 years of age. Unfortunately, studies that support the safety and effectiveness of SCIT in the elderly are not blinded. The European Academy of Allergy and Clinical Immunology advises specific immunotherapy as a relative contraindication for elderly patients [38]. The Canadian Society of Allergy and Clinical Immunology, in its guidelines for the use of immunotherapy, does not contraindicate this treatment for aged patients [39]. However, the presence of co-morbid cardiac or pulmonary conditions might increase the risk of a poor outcome following a systemic reaction. Other factors/co-morbidities to consider before starting immunotherapy in the elderly are: severe or unstable asthma, beta-adrenergic blocker and angiotensin converting enzyme inhibitors (ACEI) therapy, autoimmune diseases, and neoplastic diseases. There are potential elements of risk that can be influenced by beta-adrenergic blockers in the setting of vaccine administration. Reactions might be more frequent, more severe, and refractory to treatment [40]. Concomitant treatment with beta-adrenergic blockers does not appear to increase the risk for sistemic reaction to SCIT, but may result in more protracted and difficult to treat anaphylaxis. There is an ongoing debate on whether ACEI should be substituted prior to initiation of immunotherapy for safety reasons [41]. The medical literature reports no double blind placebo controlled (DBPC) studies specifically evaluating the efficacy of SCIT in elderly asthmatic patients. However, the prevalence of IgE-dependent allergic rhinitis and other atopic diseases in elderly patients is reportedly increasing. Based on the lack of the studies conducted to date, there is a clear need to design DBPC studies on a large scale with a significant number of patients enrolled to evaluate the efficacy and safety of the immunotherapy in the elderly; then it will be essential to confirm the results obtained in these large studies in the real-life setting [42].

In conclusion, we are in agreement with the statements reported in the “Third Update Practice Parameter on Allergen Immunotherapy” which recommend that the risk/benefit assessment for SCIT should be carefully evaluated in the elderly population because they might have co-morbid medical conditions that could increase the risks [43]. However, there is no absolute upper age limit for initiation of SCIT. Contraindications to immunotherapy in elderly patients are medical condition that reduce the patient’s ability to survive a systemic allergic reaction (i.e., patients with markedly compromised lung function), poorly controlled asthma, unstable angina, recent myocardial infarction, significant arrhythmia, and uncontrolled systemic hypertension.

#### Sublingual immunotherapy

The awareness of the efficacy and safety of allergic specific sublingual immunotherapy (SLIT) in geriatric patients is sometimes controversial. In part it is also due to the disbelief of the clinical benefits, and of the positive impact on health care costs in this age of life. In addition, it is generally known that the immune system in the elderly is down-regulated in comparison with young people and that allergies do not occur, so as not to require any more therapy. However, the reports of increased levels of total and specific IgE and consequent increase in immunological disorders are raising in literature in the last few years [44]. From the economic point of view, the impact of this treatment on the direct and indirect costs should also be outlined. Two prospective assessments of treatment in sublingual tablets for grasses, conducted in the Northern and Southern Europe, have shown that SLIT is a treatment with a favorable cost/effectiveness ratio [45,46].

Recently, it has been proved that the dendritic cells (DCs) in the elderly have an activated phenotype and an increased secretion of pro-inflammatory cytokines, which are responsible for the infiltration of eosinophils, the airway remodeling and the increase of asthma and chronic respiratory diseases [47]. In the SLIT the oral DCs are the first structures involved in the mechanism, with the aim to modulate the allergen-specific antibody responses. With particular reference to the geriatric age, compared to subcutaneous therapies it has the advantage of a home management that greatly simplifies the life of old people, who generally have difficulties in moving. In a retrospective study, Marogna et al. [48] evaluated the effect of SLIT in older patients sensitized to house-dust mite in the prevention of rhinitis and asthma progression, but also in reducing the symptoms and consumption of drugs. This was recently confirmed in a double-blind placebo-controlled study in patients over 60 years of age [49].

The safety of SLIT is confirmed by the fact that after more than 500 million doses administered to humans and 20 years of use there are no reports of fatalities; only few cases of anaphylaxis have been reported [50]. According to the Bozek et al. [49], only three patients among the elderly had local common side effects such as oral itching and facial flushing. No severe adverse reactions were observed in the active group during the study, thus confirming that SLIT is well tolerated in elderly patients. However, the compliance to SLIT may represent a major problem in real life, especially in the elderly populations.

SLIT has been demonstrated to reduce significantly both symptoms and medications, thus improving quality of life (QoL) [51]; this could be of great importance with regard to the elderly population. In fact, recent studies point out that the quality of life is significantly reduced in old people with allergic rhinitis in comparison with young people, and it can also alter the cognitive function or the mood [52].

In conclusions, SLIT is an effective and safe therapeutic option, and the only approach that can change the course of allergic respiratorydiseases; however, its use in the elderly is still not widespread. A prerequisite before starting on a SLIT, especially in the geriatric patient, is to involve the allergic patient through correct information about the various aspects of his disease: from diagnosis, pharmacological treatment options, costs, side effects, management methods, thus to verify if the patient is able to deal with the SLIT.

### Probiotics

The United Nations Food and Agricultural Organization and the World Health Organization define probiotics as “live microorganisms, which, when administered in adequate amounts, confer a health benefit to the host” [53]. Prebiotics are defined as non-digestible oligosaccharides, such as fructo-oligosaccharides and trans-beta-galacto-oligosaccharides, that selectively stimulate the growth of bifidobacteria and lactobacilli, thus producing a prebiotic effect. Synbiotics is a term referring to the use of both prebiotics and probiotics simultaneously. In taxonomy terms, the most commonly used probiotic bacteria are species of the genera Lactobacillus and Bifidobacterium. There are several mechanisms by which probiotics are proposed to exhibit beneficial effects on the host: most probably probiotics can modulate the toll-like receptors to promote TH1-cell differentiation, inhibition of antigen-induced T-cell activation and suppression of TNF [54]: the resulting stimulation of Th1 cytokines can suppress Th2 responses. A variety of human studies on the effects of probiotics administration on the management of various allergic diseases have been performed to examine the efficacy of probiotics in many allergic conditions, such as eczema and food allergy but there are limited studies on the effect of probiotic treatment for asthma that do not allow the reader to extract concrete conclusions that would be useful for everyday practice [55]. In addition, there is no study specifically addressing the use of probiotics in elderly asthmatics, and therefore no indication can be posed for this phenotype.

### Vaccinations

In older people, alterations of both innate and acquired immunity have been shown (Table 1), resulting in an increased susceptibility to infectious diseases. Viruses, including influenza, cause acute exacerbations of asthma in as many as half of adult subjects presenting to emergency rooms. Vaccines play a major role, potentially preventing or worsening of asthma symptoms. Therefore, vaccines, such as against influenza and pneumococcus, should be administered to these patients. Asthma was the most common underlying condition among old individuals hospitalized with pandemic influenza A (H1N1) infection [56]. All asthmatics should receive an influenza vaccination annually [57]. This is in accordance with the statement of the Task Force on Community Preventive Services, which recommends multicomponent interventions aimed at increasing influenza vaccination coverage [58]. In addition, asthma education for health-care professionals should include recommendations for influenza vaccination for all patients with current asthma. Although it was hypothesized that influenza vaccination may cause wheezing and adverse effect on pulmonary function, there is no significant increase in asthma exacerbations immediately after vaccination in adults or children over 3 years of age.

**Table 1:** **Immunological changes occurring in older individuals compared to younger ages**

•	Reduced number and function of hematopoietic stem cells
•	Thymic involution
•	Reduced circulating naive T cells
•	Increased frequencies of well-differentiated memory CD28− T cells with limited proliferative potential
•	Increased levels of many proinflammatory cytokines, including interleukin (IL)-6 and TNFα
•	Decreased CD4/CD8 ratios
•	Senescence of epithelial cells of the lung
•	Augmented neutrophils in the airway
•	Reduced function of eosinophils
•	Decline in the amount of macrophages and cytotoxic natural killer cells
•	Reduced capacity to stimulate antigen specific T cells of dendritic cells,
•	Reduced oxidative burst, phagocytic capacity and bactericidal activity of neutophils
•	Reduced oxidative burst and phagocytic capacity of macrophages

Respiratory bacterial infections are among the most important causes of morbidity and mortality from communicable diseases worldwide. Streptococcus pneumoniae frequently colonizes the upper respiratory tract. Local host immunity is essential to control colonizing pathogens by preventing overgrowth, spread, and invasion. Asthma is commonly considered an independent risk factor for invasive pneumococcal disease. The pneumococcal conjugate vaccine, PCV13, is currently recommended for all adults 65 years or older, in particular in older asthmatics. The 23-valent pneumococcal polysaccharide vaccine PPSV23 is also recommended for use in adults of 65 years of age who smoke cigarettes or who have asthma. Physicians and other health care professionals should therefore encourage vaccinations in elderly asthmatic patients.

### Alternative medicines

The use of Complementary/Alternative Medicines (CAMs) is an impressive emergent phenomenon in Western Countries. This widespread use is common at any age, including older people [59]. Bronchial asthma is an important field for CAM, where homeopathy, acupuncture, herbal medicines and yoga are the most utilized techniques [60]. The reasons for using CAMs usually reported by patients are: a distrust in conventional medicine, the belief that CAMs are more natural and safe, and the need for a more strict relationship with the physician [60]. Due to the large diffusion of CAM, the high prevalence of allergic diseases, and the not negligible costs, it is definitely needed that proofs of efficacy are incontrovertible [61]. Only randomized controlled trials can be suitable for the evaluation of CAM efficacy and safety. However, the vast majority of the clinical trials published up today with CAMs have a low qualitative level [62], thus making the results often difficult to interpret. On the other hand, it is claimed that “holistic” approaches cannot be standardized and submitted to rigorous study designs, because the standardization itself introduces a confounding factor [63]. Finally, it has to be considered that some of the CAM techniques are self-applied (Yoga, relaxation techniques, biofeedback) and therefore cannot be blinded.

#### Acupuncture

Acupuncture is a cornerstone of the Traditional Chinese Medicine, and is widely used for chronic illness, including asthma. The most recent reviews [64] included 11 studies with 324 participants: trial reporting was poor, and quality was judged insufficient. Indeed, looking only at those studies performed with a rigorous methodology (i.e. randomized, controlled and blinded), the effects of acupuncture are not different from the placebo treatments. Thus, the conclusion derived from meta-analysis studied and clinical trials is that acupuncture is not effective to treat asthma, although a powerful placebo effect of acupuncture as rescue medication was demonstrated [65].

#### Homeopathy

Homeopathy is based on the belief that symptoms of a disease can be cured by the same substance that provokes them, if given at ultra-dilution. Homeopathic remedies are therefore chosen according to symptoms, not to disease, and prepared with a special manual technique called “potentiation”. Homeopathy has been extensively studied in allergic diseases, and there are well-conducted and rigorous trials in both asthma and rhinitis, [66-68], but none on elderly: these studies failed to demonstrate a mesurable clinical benefit on symptoms and functional parameters in adults [66,67].

#### Phytotherapy

The traditional allopathic medicine is largely based on subtances derived from plants and herbs (e.g. theophillyne, salycilates, digitalis, morphine). The literature on herbal remedies is impressive, due to the large variety of herbs and their combinations used: *tylophora indica, boswellia serrata, pychrorryza kurroa, koleus forskholii, gynko biloba, urtica* and others. All these studies are in general of low quality, but in many cases, a clinical effect can be measured in several diseases, including bronchial asthma. This is not surprising, because most of the herbs utilized contain pharmacologically active ingredients. Positive results were obtained in rhinitis and asthma with the mixtures of herbs used in the traditional Chinese medicine, which contain ephedrine and atropine. No study has specifically addressed its use in older populations. The active ingredients may also induce undesirable side effects [69]. Moreover, at variance with proprietary marketing drugs, herbal remedies carry the risk of adulteration, incorrect collection of plants, wrong preparation and inappropriate/incorrect dosing [70]. Products containing ginseng may negatively affect the anticoagulant and hypoglycemic therapies [71], which may have dramtic consequences in older individuals. Of note, herbal remedies can be responsible for severe allergic reaction more frequently in atopic subjects [72].

#### Behavioral, physical and other complementary treatments

Physical techniques (e.g. breathing control, Yoga techniques and chiropractic/spinal manipulation) have been proposed in patients with chronic respiratory illness with the aim of improving the respiratory pattern. The majority of clinical trials of chiropractic/spinal manipulation in asthma [73,74] failed to demonstrate a clinically relevant effect. Although breathing and yoga techniques can have some effect on self-perceived well being, they cannot be recommended as an effective treatment for asthma [75]. Also behavioral techniques such as biofeedback and hypnosis have been sometimes applied in asthma, generally in low quality studies, but the overview of the literature concluded for no effect [76,77].

In conclusions, available scientific evidence does not support a role for CAM in the treatment of asthma in the elderly. The studies in the literature often have significant design flaws that weaken the conclusions such as insufficient number of patients, lack of proper controls and indadequate blinding.

### Exercise and sport

For the elderly asthmatic, exercise represents at the same time both a goal and a precious tool for treatment. On the one side, in fact, regular participation in sports and physical activity is one of the best ways for older adults, including those with chronic diseases, to promote independence, increase quality of life and improve aerobic capacity, breathing pattern, muscle strength [78]. On the other side, older asthmatics may develop a negative attitude to exercise due to a fear of symptoms occurring during or after exercise and to a lack of specific advice about exercise from specialized health professionals [79]. This lowers significantly the level of habitual activity and physical fitness, and the result is that older asthmatic are less active than their non-asthmatic peers [80]. To date, the majority of studies evaluating exercise training in asthma have been performed in children or young adults with mild-to-moderate persistent disease. Practicing any kind of sports in the elderly asthmatic must firstly consider the physiological changes in old age: loss of muscle mass; reduction in bone mass; increased percentage of fat; lower amount of body water; lack of thirst; diminishing kidney function and the very frequent presence of comorbidities, in particular related to the cardiovascular system. The regressive changes in the locomotor and the nervous system of the elderly may reduce strength, endurance, proprioceptive capacity (e.g. coordination, balance) and mobility [81]. While numerous studies deal with general physiology and sports medicine aspects in the elderly, very scant specific literature is available about asthmatic senior subjects and sports.

Exercise-induced bronchoconstriction (EIB) with- and without underlying asthma may occur also in the elderly asthmatic practicing sports, even with a higher frequency than in the general adult population taking into consideration all the comorbidities and physiological changes associated with aging previously cited. Sports with prolonged effort of more than 5–8 minutes or in cold and dry environments represent major risk factors (e.g. endurance sports, cycling, cross-country skiing). Swimming is still a controversial issue, and therefore it should be avoided for the potential risk related to chlorine byproducts inhalation. Treatment should follow the general guidelines and recommendations that apply to adults, with the limit of an arbitrary extrapolation due to the paucity of specific clinical trials in patients over 65 years. Finally, elderly athletes involved in International Association of Athletics Federations (IAAF) or in official masters’ competitions, must be aware about anti-doping regulations that apply not only to some anti-asthmatic drugs (beta-2 agonists except salbutamol, salmeterol and formoterol; systemic corticosteroids) but also to several drugs widely used to treat older adults’ comorbidities (e.g. beta-blockers, diuretics, hormones).

### Age-related and concomitant disease issues in asthma treatment

#### Adherence

The contribution of patient adherence to clinical success cannot be overestimated [82], and clinicians should always be aware of the role played by patients themselves in determining the success or failure of treatment. These general concepts apply perfectly to asthma, whose management is mainly based on the use of inhalation therapy. In the elderly, unintentional non adherence with inhalation therapy may lead to significant impairment in asthma control. Elderly patients often are affected by several chronic diseases requiring multiple medications. Complexity of the treatment can be considered a major risk factor for reduced adherence with medication. In addition, elderly patients may suffer from cognitive, hearing, or visual impairments, or other physical inabilities (such as arthritis, tremor, and low coordination) that significantly affect their ability to understand and follow treatment regimens. Despite the evidence that asthma related morbidity and mortality are higher in the elderly than in the young asthmatics, the research studies and health policies on asthma have focused mainly on children and young adults. The recently published National Institute on Aging (NIA) white paper highlighting the burden of elderly asthmatics emphasizes the need for further research to identify and intervene on factors affecting the disease in this under-studied group of patients [83]. Beliefs about treatment, such as the notion that treatment is not necessary or safe, are also correlated with decreased adherence and lower prescription refill. The beliefs are frequently held by elderly asthmatics, yet their influence on medication adherence has not been demonstrated among these patients [83,84]. In conclusion, an improvement of adherence is likely required to prevent the lack of treatment in chronic diseases, and in particular in the elderly asthmatics.

#### Comorbidities

Aging is associated with the development of numerous chronic diseases. Thus, it is quite common that elderly asthmatic subjects have additional chronic diseases, which may interfere with adherence to asthma treatment and control. Many epidemiological studies [85,86] report that, within the elderly population > 65 years, asthmatic patients would have an increased incidence of additional chronic diseases than the rest of population. Arthritis, insomnia, gastric ulcers, migraine, sinusitis, depression, cancer, and atherosclerosis were significantly more prevalent in patients with chronic airway obstruction (both asthma and COPD) according to a Dutch study [86]. In the US, Diette et al. reported that comorbid conditions, specifically COPD, heartburn, and congestive heart failure, were more prevalent in patients over 65 years of age than younger patients [87]. Depression has been reported to be associated to severe asthma in the elderly from U.S. National Heart, Lung, and Blood Institute’s Severe Asthma Research Program [88] and, in a cohort of elderly inner-city asthmatic patients, depressive symptoms were associated with poorer asthma control and quality of life, as well as with lower rates of adherence to controller medications [89].

The impact of comorbidities on asthma control of elderly asthmatics has been explored by few studies with conflicting results. According to a Canadian study [90], although more than 83% of patients with asthma who were 55 years and older reported having one or more major comorbidities, the odds of having asthma symptoms or attacks for these patients were lower than the odds in younger. On the other hand, in the same study, the odds of self-perceived health status as fair or poor were significantly higher in the older respondents with asthma (70 years and older: OR 3.10, 95% CI 2.27 – 4.12) and those with five or more comorbidities (OR 35.18, 95% CI 19.57 – 63.26). The recent findings from the Italian multicenter study on elderly asthmatics (ELSA study) showed that elderly patients with asthma associated with COPD had worse asthma control and higher rate of severe asthma exacerbation in the previous year, compared to asthmatic patients without COPD [22]. In conclusion, elderly asthmatic patients have many comorbidities, which, with the possible exclusion of COPD and depression, do not seem to have a direct impact on asthma control in patients under specialist care. Nevertheless, the comorbid conditions in the elderly patient makes the diagnosis of asthma more difficult, so that elderly asthmatic patients appears to be undertreated, with the consequence of a higher hospitalization rate and mortality.

### Asthma and concomitant rhinitis

Asthma is frequently associated with nasal/sinonasal comorbidities such as rhinitis (allergic and non-allergic) or chronic rhinosinusitis (with and without nasal polyps), and these conditions may act as aggravating factors for asthma itself. Rhinitis in the elderly seems to have peculiar clinical and cytologic characteristics [52], and the strong association with asthma seems to be confirmed [91,92]. Therefore, treating concomitant rhinitis or chronic rhinosinusitis is part of the correct and global management of asthma also in older adults.

#### Antihistamines

Antihistamines are a mainstay in the treatment of allergic respiratory diseases due to their H1 receptors antagonism. The first-generation anti H-1 (e.g. chlorpheniramine, diphenhydramine) are effective on allergic inflammation but they have well-known side effects, due to the lack of specificity for the H1 receptor [93]. Sedation, anxiety, confusion and decreased reaction time are more pronounced in the elderly, as well as the anticholinergic effects, such as drying of the mouth and eyes, blurred vision, disequilibrium, urinary retention and constipation, arrhythmias and postural hypertension. For these reasons symptomatic prostatic hypertrophy, bladder neck obstruction and narrow angle glaucoma should be taken into consideration as contraindications to the use of first-generation antihistamines [93,94]. The potential cardiac toxicity deserves some considerations. It is not a class effect and does not occur through the H1-receptor. Nevertheless it has been described that some first-generation H1-antihistamines, such as promethazine, brompheniramine, diphenhydramine in some cases are able to prolong the QT interval, and potentially cause serious polymorphic ventricular arrhythmias such as *torsades de pointes*. These effects have been observed in case of large doses of overdoses, however the potential cardiac comorbidities should be carefully assessed when prescribing an antihistamine drug [93]. The changes in body composition and the decreased activity of liver in the elderly may account for a major risk of adverse events associated with first generation H1-antihistamines use [94,95].

The second-generation of H-1 receptor antagonists (e.g. loratadine, cetirizine, fexofenadina, desloratadina, levocetirizina) provide a better safety and tolerability profile due to their low cross blood brain barrier and greater specificity for their receptor [96]. The propensity of two second generation H1-antihistamines introduced in the 1980s, astemizole and terfenadine, to exert a cardiac toxic effect has been described [93]. These two drugs are no longer approved by regulatory agencies in most countries. No or not clinically significant cardiac effects have been reported for the second-generation H1-antihistamines loratadine, fexofenadine, mizolastine, ebastine, azelastine, cetirizine, desloratadine, levocetirizine, rupatadine and bilastine [96]. Second generation antihistamines have little or no sedative or anticholinergic effect [96]. However, because of the reduced metabolic activity, treatment should be started with a lower dose in this age group. In fact some second-generation H1-antihistamines such as desloratadine, loratadine and rupatadine are metabolized by the system cytochrome P450. Cetirizine is excreted largely unchanged in the urine, and fexofenadine is excreted largely unchanged in the feces. However interactions may be more likely to be associated with first generation H1-antihistamines than second-generation H1-antihistamines, which have a wider therapeutic index [96]. Thus it is recommended to use second-generation antihistamines in the elderly.

#### Nasal corticosteroids

For many other classes of drugs, clinical trials with intranasal steroids were generally conducted in adults, typically included those 65 years and older, but without reporting data specific to this population. However, there is a consensus in considering intranasal steroids as the first-line treatment for moderate to severe allergic rhinitis in the elderly, effectively treating all symptoms of rhinitis [97]. A randomized controlled trial studied the effects of mometasone furoate nasal spray in patients older than 65 years of age suffering from perennial allergic rhinitis, showing it to be an effective treatment in this cohort [98]. An open-label trial in 18 patients 65 years and older with a history of moderate-to-severe rhinitis treated with either azelastine nasal spray 2 sprays per nostril bid (1.1 mg) or fluticasone propionate 2 sprays per nostril qd (200 mcg) for a 6-week study period showed that both treatments improved symptom scores compared to baseline, with statistically significant improvement reached earlier for fluticasone compared to azelastine, suggesting that fluticasone propionate is safe and more effective than azelastine in older adults [99]. Intranasal steroids are generally well tolerated by older patients [100]: the most common encountered side effects are mild epistaxis, dryness, burning and nasl crusting. In older adults, particular attention should be put for possible systemic side effects of intranasal steroids, such as effects on bone metabolism which may rise specific concern particularly in older and postmenopausal women, and in patients receiving steroids for other concurrent conditions such as asthma itself. However, based on the lack of significant changes in biochemical markers of bone turnover in several studies, these intranasal corticosteroids agents do not appear to be associated with reductions in bone mineral density or osteoporosis [101,102].

Another concern in using intranasal corticosteroids in older patients is the possible effect of these agents in fostering glaucoma. A case–control study of 9793 patients, age 66 or older, with a new diagnosis of borderline glaucoma, open angle glaucoma, or ocular hypertension, and 38325 controls randomly selected showed that there was no increased risk for these diseases with intranasal steroid use. The risk of ocular side effects appears to be negligible due to the low systemic bioavailability of most available intranasal steroid preparations [103]. In conclusion, intranasal corticosteroids have the most favorable safety and efficacy profiles in older individuals and therefore they should be recommended as first-line treatment of rhinitis or chronic rhinosinusitis in older patients [104].

#### Topical antihistamines

Topically administered antihistamines are clinically effective, with the advantage of delivering the medication directly to the target area. Common adverse events of inhaled antihistamines are usually mild and include bitter taste, headache, dry mouth, sedation and application site irritation. More relevant side effects, mainly related to the first generation molecules, including urinary retention, prolonged QT interval, arrhythmias and constipation are however more prevalent in elderly patients due to the commonly observed comorbidities, such as heart diseases, prostatic hypertrophy and narrow angle glaucoma. Moreover, elderly patients usually take many drugs, thus increasing the risk of interaction between medications. Therefore, the antihistamine selection for the treatment of concomitant allergic rhinitis in elderly asthmatic patients should be made carefully and topical II generation molecules should be preferred [94]. In subjects older than 65 years, azelastine has been shown to be well tolerated [105]. Furthermore, pharmacokinetic studies indicate that the systemic bioavailabilities of marketed azelastine hydrochloride nasal spray products is about 40% and that 75% of the excretion of its metabolites is through faeces while just 25% is through urines, making it safer also for patients with renal impairment [106]. Levocabastine is mainly (about 70% of the absorbed dose) excreted unchanged in the urine, and has to be therefore used with caution in patients with renal impairment. After intranasal administration, low plasma concentrations of levocabastine are reached; therefore, drug interactions are not clinically significant, excluding those with inhibitor of cytochrome P450, such as erythromycin and ketoconazole. No clinically significant mean changes from baseline in QT or QT_c_ intervals were reported in literature [107].

At last, a novel intranasal formulation, combining the second generation antihistamine azelastine hydrochloride and fluticasone propionate in a single device, has been recently developed, showing superiority over its single components [108]. Although these studies have been performed in a general adult population, we can reasonably assume the combination could be effectively and safely used also in elderly patients. Furthermore, the administration of different drugs in the same preparation could facilitate the compliance for older patients.

## Conclusions

### Final considerations and novel perspectives

The most recent research identified some promising “biological” approaches that could be successfully applied also in the elderly, and new treatment modalities have been introduced, mainly concerning biological agents or immunotherapy developments [109] (Table 2). Of note, there is no upper limit of age for the prescription of omalizumab, thus it can be used also in the elderly, provided that a clear diagnosis of IgE-mediated disease is made. The immediately following option is the anti IL-5 Mab (mepolizumab, reslizumab). This approach is mainly targeted to uncontrolled asthma with a clearly demonstrated eosinophilia, and there is, so far, no apparent contraindication to its use also in the elderly. Possible molecular targets, at least in asthma, are IL-4 and IL-13, both involved in atopy related inflammatory processes, and partly sharing the same receptor. Thus, targeting IL-4 or IL-13 alone may be ineffective because of redundant mechanisms of the two cytokines. Based on this concept, pitrakinra, an IL-4Ra double mutein involved in both IL-4 and IL-13 signalling, was developed and tested in asthma clinical trials with favourable results [110]. In addition, anti CD25 (daclizumab) and anti IL-5 (benralizumab) receptors are currently being explored [109].

**Table 2:** **The most advanced biological preparations used in asthma**

**Drug**	**Target**	**Function**
Atrakincept	Anti-IL4	Binds IL-4 thus blocking the interaction with its receptor. Reduces the proliferation of Th2 lymphocytes. Phase II
Pascolizumab		
Benralizumab	Anti IL-5 receptor	Reduces the proliferation and activation of eosinophils. Phase II
Daclizumab	Anti CD-25	Blocks the receptor for IL-2
Kerliximab	Anti CD-4	Reduces the proliferation of CD4+ lymphocytes. Phase II
Mepolizumab	Anti IL-5	Reduces the proliferation and activation of eosinophils. Phase III
Reslizumab		
Omalizumab	Anti IgE	Complexes circulating IgE and impedes their binding to receptor. Commercialized
Pitakinra	IL-4 mutein	Competes with IL-4 and IL-13 receptor. Phase III

For about one century, specific immunotherapy has been administered only subcutaneously, but in the last 20 years, the sublingual route has been accepted as a viable alternative, and its use is nowadays largely diffused [50]. This modality could represent an optimal choice in the elderly, where convenience, including the at home administration, remains the most important issue. However, clinical trials of immunotherapy in the elderly are rare and sparse, and senior subjects were enrolled only on an anecdotal way. For allergen immunotherapy, several alternative modalities are currently being developed, including the new routes of administration, the preparation of extracts and the use of adjuvants (Table 3). Within those approaches, the intralymphatic and the epicutaneous administration routes seem to be the more feasible in the immediate future, and particularly suitable for elderly, due to the non invasive or not time-consuming approach [111]. Considering that the IgE response to allergens not always declines with age, the association/combination with Th1-inducing adjuvants is a promising approach. Indeed, there are already available commercial preparations of allergens adjuvanted with bacteria-derived components, which stimulate the toll like receptor 4. The use of bacterial DNA as stimulants of the toll-like receptor 9, to be associated with allergens is an intriguing possibility [112]. Similarly, good results have been obtained with the recombinant allergenic molecules, but this approach is limited by regulatory aspects, and by the fact that a specific mixture of allergen proteins would be required [113]. In this regard, the component-resolved diagnosis would facilitate the appropriate prescription of immunotherapy, by discerning true sensitizations and cross-reactivities [114].

**Table 3:** **The recent developments of allergen immunotherapy**

**Formulation**	**Allergen**
Subcutaneous/sublingual	Recombinant
Epicutaneous	Purified
Intralymphatic	Bacteria-derived adjuvants
Micro-injection	DNA-derived adjuvants
	Allergen peptides

For long time, it was assumed that allergic respiratory disorders are typical of children, adolescents and young adults. In fact, most clinical trials were designed to include patients below 65 years of age. The time has come to face with allergic diseases in older ages, and this carries safety concerns and implications for efficacy. The management of allergic asthma in the elderly will only be successful when clinical randomized trials and observational real-life studies will be specifically conceived for older individuals.

## Abbreviations

CAMs: Complementary/alternative medicines
ICS: Inhaled corticosteroids
SABA: Short-acting beta-2 agonists
LABA: Long-acting beta-2 agonists
LTRA: Leukotriene antagonists
mAb: monoclonal antibody
SCIT: Subcutaneous immunotherapy
ACEI: Angiotensin converting enzyme inhibitors
DBPC: Double blind placebo controlled
SLIT: Sublingual immunotherapy
DC: Dendritic cell
QoL: Quality of life
EIB: Exercise-induced bronchoconstriction
IAAF: International Association of Athletics Federations
NIA: National Institute on Aging
